# Cognitive and neural strategies during control of the anterior cingulate cortex by fMRI neurofeedback in patients with schizophrenia

**DOI:** 10.3389/fnbeh.2015.00169

**Published:** 2015-06-25

**Authors:** Julia S. Cordes, Krystyna A. Mathiak, Miriam Dyck, Eliza M. Alawi, Tilman J. Gaber, Florian D. Zepf, Martin Klasen, Mikhail Zvyagintsev, Ruben C. Gur, Klaus Mathiak

**Affiliations:** ^1^Department of Psychiatry, Psychotherapy, and Psychosomatics, Medical School, RWTH Aachen UniversityAachen, Germany; ^2^JARA-Translational Brain Medicine, RWTH Aachen UniversityAachen, Germany; ^3^Clinic for Child and Adolescent Psychiatry, Psychosomatics and Psychotherapy, RWTH Aachen UniversityAachen, Germany; ^4^Department of Child and Adolescent Psychiatry, School of Psychiatry and Clinical Neurosciences and School of Paediatrics and Child Health, Faculty of Medicine, Dentistry and Health Sciences, The University of Western Australia (M561)Perth, WA, Australia; ^5^Specialised Child and Adolescent Mental Health Services (CAMHS), Department of Health in Western AustraliaPerth, WA, Australia; ^6^Department of Psychiatry, University of PennsylvaniaPhiladelphia, PA, USA; ^7^Institute of Psychiatry, Psychology and Neuroscience, King’s College LondonLondon, UK

**Keywords:** social reinforcement, cognitive therapy, psychotherapy, remediation therapy, cognitive strategies, rostral and dorsal ACC, brain computer interface, self-regulation

## Abstract

Cognitive functioning is impaired in patients with schizophrenia, leading to significant disabilities in everyday functioning. Its improvement is an important treatment target. Neurofeedback (NF) seems a promising method to address the neural dysfunctions underlying those cognitive impairments. The anterior cingulate cortex (ACC), a central hub for cognitive processing, is one of the brain regions known to be dysfunctional in schizophrenia. Here we conducted NF training based on real-time functional magnetic resonance imaging (fMRI) in patients with schizophrenia to enable them to control their ACC activity. Training was performed over 3 days in a group of 11 patients with schizophrenia and 11 healthy controls. Social feedback was provided in accordance with the evoked activity in the selected region of interest (ROI). Neural and cognitive strategies were examined off-line. Both groups learned to control the activity of their ACC but used different neural strategies: patients activated the dorsal and healthy controls the rostral subdivision. Patients mainly used imagination of music to elicit activity and the control group imagination of sports. In a stepwise regression analysis, the difference in neural control did not result from the differences in cognitive strategies but from diagnosis alone. Based on social reinforcers, patients with schizophrenia can learn to regulate localized brain activity. However, cognitive strategies and neural network location differ from healthy controls. These data emphasize that for therapeutic interventions in patients with schizophrenia compensatory strategies may emerge. Specific cognitive skills or specific dysfunctional networks should be addressed to train impaired skills. Social NF based on fMRI may be one method to accomplish precise learning targets.

## Introduction

Aspects of cognitive functioning, such as memory, attentional performance, or face recognition, are impaired in patients with schizophrenia, and can lead to significant disabilities in occupational, social, and economic functioning (see Keefe and Harvey, 2012, for a review). These particular impairments often persist throughout all stages of illness (Censits et al., 1997). Antipsychotic agents often improve positive symptoms, but treatments that improve social and cognitive functioning are still warranted (Keefe et al., 1999). Cognitive skills have become an important therapeutic target in patients with schizophrenia, and are associated with long-term outcomes and prognosis (Purdon et al., 2000). Psychological strategies, such as cognitive remediation therapy, have been introduced to improve cognitive deficits (Turkington et al., 2004) and should target different symptom domains, e.g., executive function, attentional performance, and aspects of memory (Kurtz et al., 2001). Such improvements of specific cognitive functions (Medalia and Lim, 2004) can be expected to normalize dysregulated neural activity (Kurtz, 2012; Penadés et al., 2013). Although one may expect that addressing a supposed neural dysregulation in a direct manner should lead to improvements in cognitive functions, so far, none of the existing therapeutic approaches directly addresses the underlying neural deficits (Turkington et al., 2004).

The anterior cingulate cortex (ACC) plays an important role in the pathogenesis of schizophrenia. Structural magnetic resonance imaging (MRI) and neuropathological findings demonstrate gray matter reductions of the ACC in patients with psychosis, occurring already prior to its onset and, eventually, progressing with illness duration (Fornito et al., 2009). In functional MRI (fMRI) studies, patients with schizophrenia showed reduced conflict- (Snitz et al., 2005) as well as error-related activity in the ACC (see Melcher et al., 2008, for a review; Carter et al., 2001; Alain et al., 2002; Kerns et al., 2005), which may normalize upon administration of antipsychotic medications (Snitz et al., 2005; Adams and David, 2007). Studies reported hypo-activation in patients only for the rostral division of the ACC during Stroop (Carter et al., 1997), Go/NoGo (Laurens et al., 2003), oddball (Liddle et al., 2006), or emotion recognition tasks (Habel et al., 2010). Reduced ACC activity in patients with schizophrenia plays an important role in the development of deficits in different cognitive domains, such as attention, working memory, verbal production, response monitoring, and inhibition (Sanders et al., 2002).

fMRI-based neurofeedback (NF) trains subjects to control localized brain activity (Bray et al., 2007). Using real-time fMRI, a Brain-Computer Interface (BCI) provides feedback of the momentary activity in a selected brain area (Weiskopf et al., 2004). This has been suggested as a method to modulate specific functions of neural networks (Yoo et al., 2006). Several studies have demonstrated that healthy participants can learn the control of circumscribed brain regions using fMRI-based NF (Yoo et al., 2006; Caria et al., 2007; Rota et al., 2009; Hamilton et al., 2011; Scharnowski et al., 2012; Lawrence et al., 2013). Moreover, first attempts of using fMRI-NF as a therapeutic intervention have been made (e.g., Subramanian et al., 2011). Patients with treatment-resistant depression showed clinical improvements after localized regulation (Linden et al., 2012). In patients with chronic pain, improved symptomatic control was associated with ACC regulation (deCharms et al., 2005). Moreover, successful ACC regulation was achieved in other study groups, i.e., such as patients with nicotine addiction (Li et al., 2013). In patients with schizophrenia, NF of the bilateral anterior insula activity led to an emotion recognition bias towards disgust, demonstrating that patients with schizophrenia were able to gain voluntary control over their regional brain activity (Ruiz et al., 2013). So far, however, no NF study aimed to improve cognitive functioning in patients with schizophrenia. The ACC seems a promising target region for such an intervention, because the ACC is considered to be a central hub for cognitive processing. In particular, in patients with schizophrenia changes in ACC functioning were associated with impaired responses in cognitive tasks, especially in a Stroop cognitive interference task (Minzenberg et al., 2009). Voluntary control of ACC function may help to overcome specific cognitive deficits, and the applied cognitive strategies may provide a significant pathway to improve cognitive skills in this particular disorder (Weiskopf et al., 2004), even over a longer period of time (Harmelech et al., 2013).

In patients with schizophrenia, the voluntary regulation of ACC function may influence circumscribed cognitive dysfunctions, and thereby may contribute to the understanding of cognitive remediation therapies. The present study applied fMRI-based NF over 3 days to train volitional control of the ACC in participants with schizophrenia and in healthy controls. Since this particular brain area underlies cognitive dysfunctions in the patient group, we expected differences between the groups with respect to the neural pattern of activation as well as the applied cognitive strategies. Therefore, reported strategies were content-coded and related to the activation pattern during ACC regulation. We expected different frequencies of emerging content categories between the groups. Further, we explored correlations of regulation amplitudes with symptom and mood scales.

## Materials and Methods

### Participants

We investigated 11 patients with a confirmed diagnosis of schizophrenia (five females) with a mean age of 38.9 ± 9.3 years, and an age- and gender-matched control group of 11 healthy subjects (see Table 1). Patients were recruited through the Department of Psychiatry, Psychotherapy and Psychosomatics of the University Hospital Aachen. All of the patients were diagnosed with schizophrenia according to the Diagnostic and Statistical Manual of Mental Disorders (DSM-IV) criteria, and all of them were medicated with a stable dose of antipsychotics (seven with a single and four with a combination of two). The diagnoses were ascertained by experienced clinicians using the German version of Structured Clinical Interview for the DSM-IV (SKID; Wittchen et al., 1996). Patients with acute psychiatric or any neurological co-morbidity were excluded. The control group comprised 11 healthy subjects, matched for age (38.9 ± 9.3 years) and gender (five females), and was recruited via public advertisement. All but one participant were right-handed as rated with the Edinburgh Handedness Inventory (Oldfield, 1971; see Table 1) and gave written informed consent to the experimental protocol, which was in accordance with the Helsinki Declaration and approved by the Ethics Committee of the RWTH Aachen University, Germany.

**Table 1:** **Demographic and clinical characteristics of schizophrenia (*n* = 11) and control group (*n* = 11)**.

Subject	Gender	Age	Handedness	Smoker	Education	Initial PANAS (pos)	Initial PANAS (neg)	QMI score	Manifestation age	Psychiatric comorbidity	Medication (anti-psychotic, anti-depressiv)	Initial score
Patient 1	Female	35	Right		High School	30	10	25	17	–	Ziprasidone	39
Patient 2	Female	46	Right		University	29	13	23	43	–	Aripiprazole, clozapine, venlafaxine	61
Patient 3	Female	44	Right		College	31	10	27	33	–	Quetiapine, amisulpride, escitalopram	41
Patient 4	Male	47	Right		University	27	11	24	39	–	Quetiapine	50
Patient 5	Male	39	Right	✓	College	29	10	19	24	F63.0	Aripiprazole, clozapine, citalopram	57
Patient 6	Male	22	Right		University	18	16	20	21	F20.4	Quetiapine	39
Patient 7	Female	50	Right	(✓)	College	29	10	32	32	–	Quetiapine	49
Patient 8	Male	28	Right	✓	High School	31	11	10	17	F19.1	Amisulpride, dipiperone	51
Patient 9	Male	50	Right	✓	High School	27	11	30	19	–	Risperidonw, promethazine	50
Patient 10	Female	35	Right	✓	High School	20	12	35	22	F63.0	Olanzapine, promethazine	42
Patient 11	Male	32	Right	✓	University	27	11	25	29	–	Aripiprazole, clozapine	58
Control 1	Female	52	Right		College	27	10	33
Control 2	Male	43	Right		High School	24	10	33
Control 3	Female	49	Right		University	30	10	17
Control 4	Female	53	Right	(✓)	Elementary School	31	15	32
Control 5	Male	59	Right		University	38	10	34
Control 6	Male	43	Right	✓	University
Control 7	Male	22	Right		University	35	11	24
Control 8	Male	26	Right	(✓)	University	24	14	29
Control 9	Male	26	Right		University	32	16	24
Control 10	Female	31	Right		University	27	18
Control 11	Male	23	Left		University	29	12	20

*PANAS: Positive and Negative Syndrom Scale; QMI: Questionnaire upon Mental Imagery; PANSS: Positive and Negative Syndrom Scale (✓): currently abstinent from smoking F63.0: pathological gambling, F20.4: post-schizophrenic depression, F19.1: multiple drug abuse*.

### Procedure

**Figure 1:**
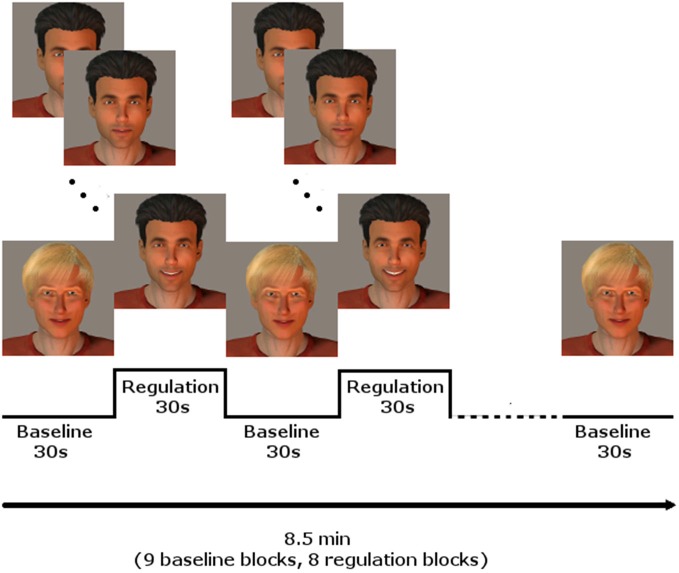
**NF paradigm using social reward**. In blocks of 30 s, the dark-haired avatar gave feedback of localized brain activity by smiling with rising intensity, whereas the fair-haired face instructed to count backwards, serving as baseline condition. Baseline and regulation blocks summed up to 8.5 min for one run. Three runs were conducted on each of the three training days.

Every participant underwent three NF training sessions on three different days during 1 week, with at least 1 day without training in between. Each session included three NF runs. The runs comprised eight regulation and nine baseline blocks, lasting 30 s each, starting and ending with a baseline block, resulting in 8.5 min per run. During the regulation blocks, the momentary BOLD activation was fed back to the participants via a BCI providing social rewards (Figure 1; for details see Mathiak et al., 2010): In short, the avatar of a dark-haired male human (created using Poser Pro, Smith Micro, Inc., USA) smiled at the participants with rising intensity when the activity of the ACC increased. In contrast, it gradually returned to a neutral expression when the activity decreased. A fair-haired, slightly smiling avatar indicated the baseline condition, instructing to count backwards from 100 in steps of three. The association of dark and fair hair with regulation and baseline condition were randomized and counterbalanced across subjects.

The Positive and Negative Affect Scale (PANAS; Watson et al., 1988) assessed mood before and after each of the three NF sessions, i.e., a pre and a post value for each day. After the experiment, imagination abilities were assessed with the short version of Betts’ Questionnaire upon Mental Imagery (QMI; Sheehan, 1967). Patients’ clinical status was assessed using the Positive and Negative Syndrome Scale (PANSS; Kay et al., 1987).

### Instructions and Strategies

During NF blocks, subjects were advised to up-regulate the smile intensity (feedback signal) using a personalized individual mental strategy. They were provided with a standardized protocol containing information on the hemodynamic delay and the instruction to only switch in between different strategies after trying one for at least 10 s. Some template strategies from different cognitive domains were named, i.e., positive autobiographic memories, picturing oneself doing sports or playing an instrument, and concentrating on certain perceptions like feeling the temperature of one’s own left foot. However, it was clarified that the subjects needed to find individual ways and strategies to achieve successful regulation of the feedback signal, and that they would be asked to report what kind of strategies they applied after each feedback run as well as in an interview at the end of every training day. Further, they rated their perceived control success over the feedback signal on a scale from 1 to 10. During baseline blocks, subjects were instructed to count backwards in steps of three, starting with 100. They were advised to concentrate on counting and not to think about anything else.

### Analysis of Cognitive Strategies

The reported strategies were content-coded by three independent raters with the categories *Music* (German: “Musik”), *Sports* (“Sport”), *Mention of other people* (“Gedanken, in deren Formulierung explizit andere Menschen erwähnt werden”), and *Others* (“Sonstiges”). The system yielded an inter-rater reliability of α = 0.85 (Krippendorffś Alpha Reliability Estimate; Krippendorff, 1970). Two-sample *t*-tests compared the frequencies of the four categories between the groups. Furthermore, we conducted a stepwise regression analysis for the dorsal and the rostral ACC peak activity to analyze contributions of cognitive strategies to the neural activity.

### Data Acquisition

Imaging was performed on a 3T MRI scanner (Magnetom TRIO, Siemens medical systems, Erlangen, Germany). Sixteen transverse slices were acquired in parallel to the anterior commissure—posterior commissure (AC-PC) plane with echo planar imaging (EPI) at a repetition time TR = 1 s (echo time TE = 28 ms; matrix size = 64 × 64 with 3 × 3 mm^2^ in-plane resolution; slice thickness = 3 mm; gap = 0.75 mm; flip angle = 67°). Each NF run comprised 520 volumes. For optimal and reproducible ACC coverage, the slices were positioned so that the AC-PC plane was between the 7th and the 8th slice from the bottom. Additionally, we collected a T1-weighted structural scan for superposition of functional maps and brain anatomy (MPRAGE, field of view FOV = 256 × 256 mm, 176 sagittal slices, 1 mm^3^ isotropic voxels, TR = 1900 ms, TE = 2.52 ms, flip angle = 8°).

### On-Line Data Analysis

An observer-independent anatomical template of the ACC determined the target region. In particular, it represented the cingulate cortex from the Automated Anatomical Labeling (AAL) atlas restricted to the quadrant with *y* and *z* coordinates greater than zero in the Montreal Neurological Institute (MNI) space. The individual images were realigned to a reference male or female EPI dataset and image raw data was extracted at and averaged over the predefined mask. First, exponential moving average algorithm removed low-frequency drift. Second, outliers, and high-frequency fluctuations were reduced with a modified Kalman filter (see Koush et al., 2012, for details). The data were scaled as to 1% signal change represented the full range of the display from the mild smile to the full smile of the avatars face. The baseline periods achieved a return to the initial signal level due to the drift removal. Data processing and feedback visualization were performed using Matlab 7.7 (The Mathworks, Natick, MA, USA).

### Off-Line Data Analysis

Statistical parametric mapping (SPM8)^1^ was conducted following standard procedures with realignment for motion correction, normalization into the MNI template space (Collins et al., 1994), and spatial smoothing with 8 mm full-width at half-maximum Gaussian kernel. Blocks for regulation conditions entered into a general linear model convolved with hemodynamic response function as independent variables. Second-level statistics conducted a group comparison using a two-sample *t*-test. After model estimation, a mask was applied including only voxels within the ACC. We used the WFU Pickatlas^2^ to define the ACC mask, taking the provided anatomical template of the cingulate cortex with the *y* coordinate ≥ 0. Levels of significance were set according to *p* < 0.05 after family-wise error correction (FWE).

A probit model tested whether there was predictive power of categories on the group variable. *Post hoc t*-tests identified the contributions of each category. Further, individual contrast estimates from dorsal and rostral subdivisions of the ACC entered linear regression analysis with group indicator, test scores, and category of strategy as predictors. Stepwise regression analysis investigated whether the subsequent addition predictors added to the explanatory power of the hierarchical models more than chance effects. The level of statistical significance was set according to *p* < 0.05 without correction for multiple testing owing to the exploratory character.

## Results

### ACC Regulation

**Figure 2:**
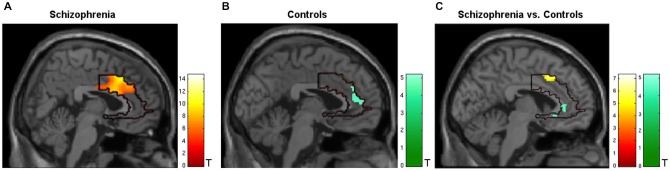
**Activation during regulation of the ACC (outlined ROI). (A)** Group of patients with schizophrenia (*n* = 11, warm colors), **(B)** matched controls (*n* = 11; cold colors), **(C)** patients > controls (warm) and controls > patients (cold). Across all training sessions and days, the patients activated the dorsal part of ACC whereas the control group yielded regulation at the rostral subdivision to change the signal from the allover ROI (*p* < 0.05, FWE- corrected).

During regulation as compared to the counting baseline blocks the patients yielded significant increase in activation in the* a priori* defined ACC mask (*T*_peak_ = 14.75, *p* < 0.05, FWE-corrected). The bilateral activation cluster was located in the dorsal subdivision of the ACC (Brodmann area [BA] 24, and partly in BA 33; Figure 2A, see Table 2). The control group up-regulated the ACC as well, but activated the rostral subdivision (*T*_peak_ = 5.19, *p* < 0.05, FWE-corrected; BA 32 and 33; Figure 2B; Table 2). The difference in localization was confirmed within a direct comparison: the patient group exhibited a greater activation of the dorsal subdivision (*T*_peak_ = 7.32, *p* < 0.05, FWE-corrected), whereas the control group yielded stronger rostral subdivision activity (*T*_peak_ = 5.00, *p* < 0.05, FWE-corrected; Figure 2C). These findings were located within the* a priori* ACC mask, but also yielded significance without small volume correction. The explorative correlations between brain activity at the cluster peaks and scales for mood, imagery, or symptom severity (average pre- and post-PANAS, QMI, PANSS) yielded no significant brain-behavior relationships at this level (all *p* > 0.1).

**Table 2:** **Activation of the ACC during neurofeedback**.

Contrast	Subdivision of ACC	Brodmann area	*T*_peak_	MNI Coordinates (*x, y, z*)	Cluster size (μl)
Pat	Dorsal	24	14.75	14, 16, 34	18.68
Cntl	Rostral	32, 33	5.19	−4, 38, 28	0.35
Pat > Cntl	Dorsal	24	7.32	−8, 14, 44	1448.00
Cntl > Pat	Rostral	32	5.00	2, 36, 0	0.17

*Cluster size refers to the whole brain analysis without ACC mask. In the “Pat > Cntl” contrast, the large volume reflects the confluence with a distributed pattern. Pat: patients; Cntl: controls*.

### Whole-Brain Analysis

Exploratory mapping analysis of the entire volume indicated significantly higher activation during regulation blocks for patients with schizophrenia vs. controls in bilateral superior temporal gyri (*T_peak_* = 8.60, *p* < 0.05, FWE-corrected), rolandic opercula (*T*_peak_ = 6.79, *p* < 0.05, FWE-corrected), pre- (*T*_peak_ = 6.50, *p* < 0.05, FWE-corrected) and postcentral gyri (*T*_peak_ = 6.63, *p* < 0.05, FWE-corrected), as well as in the left middle temporal gyrus (*T*_peak_ = 7.22, *p* < 0.05, FWE-corrected) and the left inferior parietal gyrus (*T*_peak_ = 6.49, *p* < 0.05, FWE-corrected). The reversed contrast revealed lower activity in the right supramarginal gyrus (*T*_peak_ = 8.30, *p* < 0.05, FWE-corrected) and the right middle temporal gyrus for the patient group (*T*_peak_ = 6.43, *p* < 0.05, FWE-corrected).

### Cognitive Strategies

**Figure 3:**
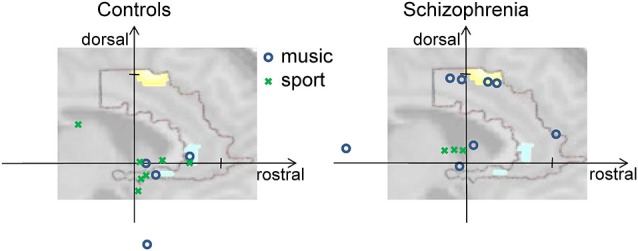
**Activity at rostral and dorsal ACC clusters**. The vertical axis shows individual activation amplitudes in the dorsal cluster and the horizontal axis in the rostral cluster (arbitrary units but same scaling for both axes). The background picture merely illustrates this relationship; it depicts the dorsal and rostral ACC clusters from Figure 2C. In the left panel for the control group, the data points are closer to the rostral cluster at the right side; on the right panel for the group of patients with schizophrenia, the data points tend towards the dorsal cluster positioned at the top. Further, color and form of the data points reveal the prominent strategy of the individual for the given regulation success (see insert). Cognitive strategies involving music (blue circles) were mostly used by patients and led in some of them but not in controls to high dorsal activation. Strategies mentioning sports (green crosses) were more frequently found in the controls.

All subjects reported a feeling of control over their ACC activity with a mean rating of 5.65 ± 1.35 (first day), 6.52 ± 1.33 (second day), and 6.76 ± 1.14 (third day) on a scale from 1 to 10. The strategies reported by patients and healthy controls ranged widely. For instance, subjects reported they applied thoughts on “my favorite music, songs from ABBA, Smokie, and The Sweet, on “being at the beach with my family, and on “playing soccer.” Based on the list of all reported strategies, an inductive coding scheme was derived, similar to a previous method for fMRI analysis (Mathiak and Weber, 2006). The induction and the coding were conducted blind to the diagnosis, i.e., from the list of verbatim reports without information on the respective participant. In the team of the authors, a list of potential categories was created reflecting the previous experience on reported control strategies. These initially 13 categories were iteratively fused to yield a sufficient frequency of items and an inter-rater reliability above 0.8 from two independent coders. In the final content coding scheme with four categories, the reported strategies differed between the diagnostic groups (probit model, *χ*^2^_(4)_ = 10.8, *p* < 0.05). The frequencies of *Music* and *Sports* differed significantly between the groups: while *Music* was used by eight patients and only four controls, *Sports* was applied by only three patients, but seven controls (*Music*: *T* = 2.05, *p* = 0.045; *Sports*: *T* = −2.22, *p* = 0.03; see Figure 3). Likewise, the contrast *Music—Sports* yielded a significant group difference (*T* = 2.91, *p* = 0.005). In contrast, no differences emerged for *Mention of others* (seven patients, six controls) and *Others* (10 patients, nine controls; all *p* > 0.2).

In a stepwise regression analysis, we investigated which variable contributed to the ACC signals in an independent manner. Dorsal and rostral ACC signals were extracted from the two clusters in the group comparison, rendering the group effect trivial. Indeed, in the dorsal subdivision, the group factor “diagnosis of schizophrenia” contributed to the model (*T* = 4.62, *p* < 0.0001). The group factor was also the most important predictor for rostral ACC activity as well (*T* = −3.48, *p* < 0.001); however, the strategies which were coded as *Mention of others* added slightly but significantly to the explained variance (*T* = 2.11, *p* = 0.039). Importantly, none of the other strategies was included by the stepwise regression procedure (all *p* > 0.2); in particular *Sports* and *Music* had no effect on ACC after correction for diagnosis (all *p* > 0.2). Finally, also linear learning over the days of assessment seemed not to contribute with regards to additionally explained variance in the regression model (all *p* > 0.2). Only in an exploratory repeated-measures ANOVA, regulation amplitude in the rACC changed significantly over days (*F*_(2,40)_ = 3.64, *p* = 0.035); in the dACC, only a trend emerged (*F*_(2,40)_ = 2.73, *p* = 0.078).

## Discussion

fMRI NF training of ACC activity in patients with schizophrenia led to activation of the dorsal ACC subsection, whereas controls activated the rostral subsection. In addition, different cognitive strategies were reported, i.e., related to music in patients with schizophrenia and to sports in healthy controls. The difference in strategies, however, did not contribute to the difference in neural activation. One can assume that with regards to the human body one choses pragmatically the most readily available strategy to increase reward intensity or probability, i.e., the smile on the avatar face. This may circumvent addressing the desired neural target, for instance, the rostral ACC in the group of patients with schizophrenia since it is easier for them to achieve control via signal changes in the dorsal ACC. Therefore, targets in the neural and cognitive domain need to be specific for normalizing dysfunctions in patients with schizophrenia.

The ACC can be divided into subdivisions in different ways, such as anatomically as well as functionally (Bush et al., 2000). In particular, differentiation between the rostral and the dorsal part of the ACC is of significant importance. If one of these parts is highly recruited, the other one tends to be suppressed with regards to related brain activity (Drevets and Raichle, 1998). The rostral ACC is mainly activated within emotional processes, whereas the dorsal part largely engages in cognitive tasks (see Bush et al., 2000, for a review). In our study, patients and controls performed identical tasks but activated different sub-regions of the ACC. Indeed, various studies have suggested that ACC dysfunction in patients with schizophrenia is primarily caused by impairments of the rostral subdivision of the ACC (Carter et al., 1997; Laurens et al., 2003; Liddle et al., 2006; Habel et al., 2010). Therefore, in order to achieve control over the global ACC signal, patients may have activated the dorsal part in particular. As compared to controls, in the schizophrenia group activity of the rostral part was even down regulated during feedback blocks. This finding is in line with other models suggesting an antagonistic action of both ACC subdivisions (Drevets and Raichle, 1998) but may counteract the intention of a global and thus also rostral activation increase in a therapeutic setting. This dysbalance may additionally contribute to the psychopathology of patients with schizophrenia. Thus, in particular in patients with schizophrenia, NF training with the aim to increase activity in the rostral ACC should consider to apply specific masks that do not cover the dorsal ACC.

Similar to the found neural strategies for achieving regulatory ACC control, the groups reported different cognitive strategies. The higher rate of music in the patients and sports in the controls, however, did not explain the neural pattern implying higher cognitive processing in patients with schizophrenia as compared to emotional processing in the healthy controls. Indeed, these particular categories predicted neither cognitive nor emotional ACC activity. Conceivably, deficits in motor activity and thus motor imagery may have been compensated by the patients using everyday experiences such as music (compare Zvyagintsev et al., 2013). The patients in our group of patients with schizophrenia were able to achieve a functional recovery in terms of effective regulation of the ACC signal by compensation from domains which seem to be less impaired (cognitive processing, music imagery). They did not apply the neural and cognitive strategies as expected from the healthy control group.

### Social Feedback

In contrast to most previous fMRI-based NF studies, we employed a feedback with direct social reward (Mathiak et al., 2015). Previously, the satisfaction of completing the task served as a reward in the operant conditioning procedure (Sitaram et al., 2007). A social context was found to enhance the reward value in NF (Goebel et al., 2004). The direct reward from the smiling face may reduce the influence of context, making the reward value more comparable in the group of patients with schizophrenia. Admittedly, emotion recognition deficits have been reported in patients with schizophrenia but hardly extend to smiling facial expressions (Kohler et al., 2003). Unimpaired recognition of happy emotional expression has been even documented in avatar faces; the unambiguous smile is expected as rewarding for the patients as for the controls (Dyck et al., 2010). In summary, direct reward using facial expressions may enhance the conditioning procedure and, thus, the long-term transfer improving therapeutic benefits in patients (compare Rathod and Turkington, 2005).

### Therapeutic Implications

Cognitive deficits are directly associated with impaired social functioning in patients with schizophrenia (Addington and Addington, 1999; Dickerson et al., 1999), and their improvement is of particular relevance for the course of the disorder. Even though pharmacotherapy is the major cornerstone with regard to the management of positive symptoms in patients with acute schizophrenia, it does not improve residual cognitive impairments (Keefe et al., 1999). Cognitive behavioral therapy was found to be effective in patients with schizophrenia (Pilling et al., 2002b; Rector and Beck, 2012), even as performed within brief interventions (Turkington et al., 2002) as well as in long-term follow-ups (Sensky et al., 2000; Gould et al., 2001). These approaches, however, aimed at positive symptoms, depression, and overall symptoms only (Turkington et al., 2004). Cognitive remediation therapy targets circumscribed cognitive functions and thus should improve the daily functioning of persons suffering from schizophrenia (Medalia and Lim, 2004), but its effectiveness is still unclear [see reviews in Pilling et al., 2002a; and the (National Institute for Clinical Excellence, 2002)]. Indeed, cognitive training may lead to the application of compensatory strategies only but may not enhance the specifically targeted processes. fMRI-based NF of localized brain activity creates the possibility to directly influence specific neural networks and simultaneously control for the achieved effects.

### Limitations

All of the patients were on a stable dose of antipsychotics (seven with a single pharmacological agent, and four with a combination of two mostly atypical substances, see Table 1). These drugs may block dopaminergic pathways as well as inhibit the ACC (Holcomb et al., 1996; Miller et al., 1997), which is the area with the highest dopaminergic innervations in the cerebral cortex of primates (Paus, 2001). Nevertheless, normalization of ACC activity was observed over middle- (4 weeks; Snitz et al., 2005) and long-term (more than 6 months) treatments with atypical antipsychotics (Braus et al., 2002). Furthermore, responses to social reward were unaltered after a single dose of an atypical antipsychotic (Klasen et al., 2013). Finally, cognitive therapy usually addressed patients on antipsychotic medications, and therefore this naturalistic sample may be more relevant to study than a group of unmedicated patients. In addition, the present sample is probably closer to the reality faced by clinicians in charge of treatment of patients with schizophrenia.

The content coding scheme used here was not only developed based on previous experiences, but also on the explicitly reported strategies from the current group. However, during this process the subject information was removed from the reports. Thus, the group differences may not be explained by an observer bias for the content code. Indeed, the choice of strategies may be influenced by the sample strategies from the standardized instruction during the introduction. However, the high contributions of the social (“Mention of others”) and the rest categories as well as reports of passive music imagery indicate that the development of independent strategies was possible to the participants. Future studies may attempt to make NF-naïve subjects learning ACC control without examples for explicit cognitive strategies. However, our informal experience was that in such a setting it may take a long time for some subjects to learn regulatory control, and a relevant proportion may actually never achieve to control their respective ACC activity. Therefore, we decided to facilitate the operant condition by providing* a priori* cognitive models.

As concerns the cognitive processes and neural activation pattern during regulation, the current study did not address the allover pattern of brain activations. Thereby, it remains elusive whether the observed activation is causal for the ACC regulation or merely epiphenomena. The limited volume coverage enabled fast acquisition with a TR of 1 s However, motor areas are not fully covered rendering connectivity analyses on this dataset. Dysconnectivity can be considered core pathology in schizophrenia (Friston, 2005) and therefore the effect on connectivity self-regulation may be an important research question for future studies.

The number of patients in our NF study group is low, so that the present sample possibly underlies a selection bias, and no long-term effects have been investigated. fMRI-based NF is a demanding technique. We pointed out to the participants that the regulation was voluntary. However, we cannot exclude that particular patients suffering from schizophrenia may be suspicious with respect to techniques intending to modulate brain activity, in particular with regard to some possible paranoid symptoms and features. Furthermore, to remain attentive to the paradigm over three consecutive runs on three different days and to adhere to the experimental protocol requires a certain degree of cognitive resources, which can be compromised in acutely ill patients when compared to healthy controls. Within the restrictions of such a pilot study, the clear dichotomy between neural and cognitive strategies suggests the unmasking of fundamentally impaired processes in these patients with schizophrenia.

In contrast to some previous studies (e.g., Yoo et al., 2006), the current study did not address the question whether subject learn voluntary regulation of the ACC. Indeed, a control group without effective NF or a good enough control condition would be required to address this question. However, this would render the study significantly more ambitious with the need for twice as many patients. Further, we do not directly suggest a therapeutic application of this technique. However, NF can be expected to address specific dysfunctions and symptoms rather than broad disease categories, e.g., auditory verbal hallucinations (cf. McCarthy-Jones, 2012). Therefore, such clusters should be selected and individual neural networks addressed that can be expected to underlie or to influence the symptomatology. Nevertheless, schizophrenia is characterized by complex and individual profiles of neural dysfunctions in addition to neuroadaptive and pharmacological effects (Gaebler et al., 2015). We suggest performing detailed studies on NF learning and potential target networks in well-characterized individuals may be an important step to gather sufficient information before conducting targeted randomized controlled trials (RCTs) with sufficient likelihood to show relevant clinical effects.

## Conclusion

In the present study, patients with schizophrenia learned to regulate the activity of their ACC in response to social NF. NF may target neural dysregulation underlying some cognitive impairments in patients with schizophrenia. The observation of divergent neurophysiology and cognition during social NF in this particular disorder emphasizes the need for orientating therapeutic interventions to specific impaired functions in patients with schizophrenia.

## Author Contributions

JSC: Data acquisition, data analysis and interpretation. Manuscript writing;
KAM: Development of study paradigm and data analysis. Supervision over data analysis and interpretation. Revising the manuscript;
MD: Contribution to design and data analysis. Revising the manuscript;
EMA: Contributions to data collection and statistical analysis. Revising the manuscript;
TJG: Contribution to design and data collection. Revising the manuscript;
FDZ: Contribution to design. Revising the manuscript;
MK: Contribution to data acquisition. Revising the manuscript;
MZ: MRI support and technical support by data acquisition. Revising the manuscript;
RG: Conceptual supervision. Revising the manuscript;
KM: Supervision of and conceptual contributions to study. Data analysis and interpretation. Revising the manuscript;

All authors read and approved the final version of the manuscript. All authors agree to be accountable for all aspects of the work in ensuring that questions related to the accuracy or integrity of any part of the work are appropriately investigated and resolved.

## Conflict of Interest Statement

The authors declare that the research was conducted in the absence of any commercial or financial relationships that could be construed as a potential conflict of interest.

## Footnotes

^1^http://www.fil.ion.ucl.ac.uk

^2^http://fmri.wfubmc.edu/software/PickAtlas

## Abbreviations

ACC: anterior cingulate cortex
BCI: brain-computer interface
EEG: electroencephalography
EPI: echo planar image
NF: neurofeedback
ROI: region of interest
rt-fMRI: real-time functional magnetic resonance imaging
SPM: Statistical Parametric Mapping.
